# Intestinal Neurod1 expression impairs paneth cell differentiation and promotes enteroendocrine lineage specification

**DOI:** 10.1038/s41598-019-55292-7

**Published:** 2019-12-20

**Authors:** Hui Joyce Li, Subir K. Ray, Ning Pan, Jody Haigh, Bernd Fritzsch, Andrew B. Leiter

**Affiliations:** 1 Division of Gastroenterology, Department of Medicine, University of Massachusetts Medical School, 364 Plantation Street, Worcester, MA 01605 USA; 2 Department of Biology, University of Iowa, Iowa City, IA 52242 USA; 3 Department of Biomedical, Molecular Biology, Ghent University, Ghent, Belgium; 4 Decibel Pharmaceutical, Boston, MA USA

**Keywords:** Differentiation, Endocrine system and metabolic diseases

## Abstract

Transcription factor Neurod1 is required for enteroendocrine progenitor differentiation and maturation. Several earlier studies indicated that ectopic expression of Neurod1 converted non- neuronal cells into neurons. However, the functional consequence of ectopic Neurod1 expression has not been examined in the GI tract, and it is not known whether Neurod1 can similarly switch cell fates in the intestine. We generated a mouse line that would enable us to conditionally express Neurod1 in intestinal epithelial cells at different stages of differentiation. Forced expression of Neurod1 throughout intestinal epithelium increased the number of EECs as well as the expression of EE specific transcription factors and hormones. Furthermore, we observed a substantial reduction of Paneth cell marker expression, although the expressions of enterocyte-, tuft- and goblet-cell specific markers are largely not affected. Our earlier study indicated that Neurog3+ progenitor cells give rise to not only EECs but also Goblet and Paneth cells. Here we show that the conditional expression of Neurod1 restricts Neurog3+ progenitors to adopt Paneth cell fate, and promotes more pronounced EE cell differentiation, while such effects are not seen in more differentiated Neurod1+ cells. Together, our data suggest that forced expression of Neurod1 programs intestinal epithelial cells more towards an EE cell fate at the expense of the Paneth cell lineage and the effect ceases as cells mature to EE cells.

## Introduction

Intestinal epithelial cells are divided into two main categories - absorptive cells and secretory cells. The absorptive cells include enterocytes and the secretory cells include enteroendocrine (EE), goblet, Paneth, and tuft cells^1,2^. All five cell types are derived from common multipotent intestinal stem cells. The expression of the atonal basic helix-loop-helix (bHLH) transcription factor 1 (Atoh1) initiates the differentiation of progenitor cells that are subsequently either fated to EE cell lineages by the downstream transcription factor, Neurogenin-3 (*Neurog3, Ngn3*), or to Goblet and Paneth cell lineages upon expression of the transcriptional repressor, growth factor independent-1(*Gfi1*)^3–7^. The differentiation of Tuft cells, on the other hand, adopts an Atoh1-independent pathway involving high expression of Sox4^8,9^. EE cells are hormone-producing cells scattered individually throughout the mammalian gastrointestinal tract. These cells comprise less than 2% of the total cell population. The maturation and subtype specification of EE cells along crypt-villus axis require subsequent expression of a number of pro-endocrine transcription factors including *Neurod1*, *Lmx1a*, *Nkx2-2*, *Insm1*, *Arx*, *Isl1* and *Pax4/6*^10–19^.

The expression of Neurod1, a member of the bHLH family of transcription factors, is restricted to pancreatic islets, intestinal endocrine, stomach and neuronal cells. It regulates the expression of the insulin gene in pancreatic beta cells^20,21^, the secretin gene in small intestine^22^, and is capable to reprogram fibroblasts to a neuronal fate^23–25^ or Schwann cells into neurons^26^. In addition, Neurod1 (Nd1) also functions in governing cell cycle exit in photoreceptor lineage^27^ and can cause cell cycle arrest in *in vitro* cell culture^28,29^. Studies of Neurod1 targeted deletion mutants revealed its important functions in development and maintenance in several developing systems including the central nervous system^30–33^, the peripheral nervous systems (including the inner ear)^34–37^, the EE cells of the GI tract^7,38,39^, as well as the beta cells of the pancreas^21,40^. Neurod1 mutations have been found to cause maturity-onset diabetes of the young and late-onset diabetes (Online Mendelian Inheritance in Man 606394)^41^. Recently, Neurod1 null mutations have been linked to ophthalmological phenotypes in humans^42^.

Neurod1 is a direct transcriptional target of Neurog3 in intestinal EECs^43,44^. Early studies have shown that Neurog3 is sufficient to induce an endocrine program when expressed in the intestine of transgenic mice^6^. Recent single-cell RNA profiling of individual intestinal epithelial cells categorized both Neurog3 and Neurod1 as markers for immature^45^ or differentiating^46^ EEC precursors, suggesting a function of Neurod1 in early EEC differentiation. Another function of Neurod1 is its ability to reprogram other cell types into neurons through altering chromatin and transcription factor landscapes^47^. These results prompted us to investigate if expression of Neurod1 prior to Neurog3 can override the default function of Neurog3 in EE cell differentiation. To examine, we generated a Rosa26 Loxp-Stop-Loxp-Neurod1 (^LSL^Neurod1) mouse line that allows conditional Neurod1 expression in Villin + cells (prior to Neurog3) by crossing with Vil-cre mice or Neurog3 + cells (concomitant with Neurog3) by crossing with Neurog3-cre mice. In this manuscript, we provide data demonstrating that conditional gain-of-function of Neurod1 prior to Neurog3 expression robustly increases EE cell numbers and this differentiation process is limited to a subset of the gut progenitor cells that are competent to adopt an endocrine fate.

## Results

### Conditional expression of Neurod1 in the intestinal epithelial cells

**Figure 1:**
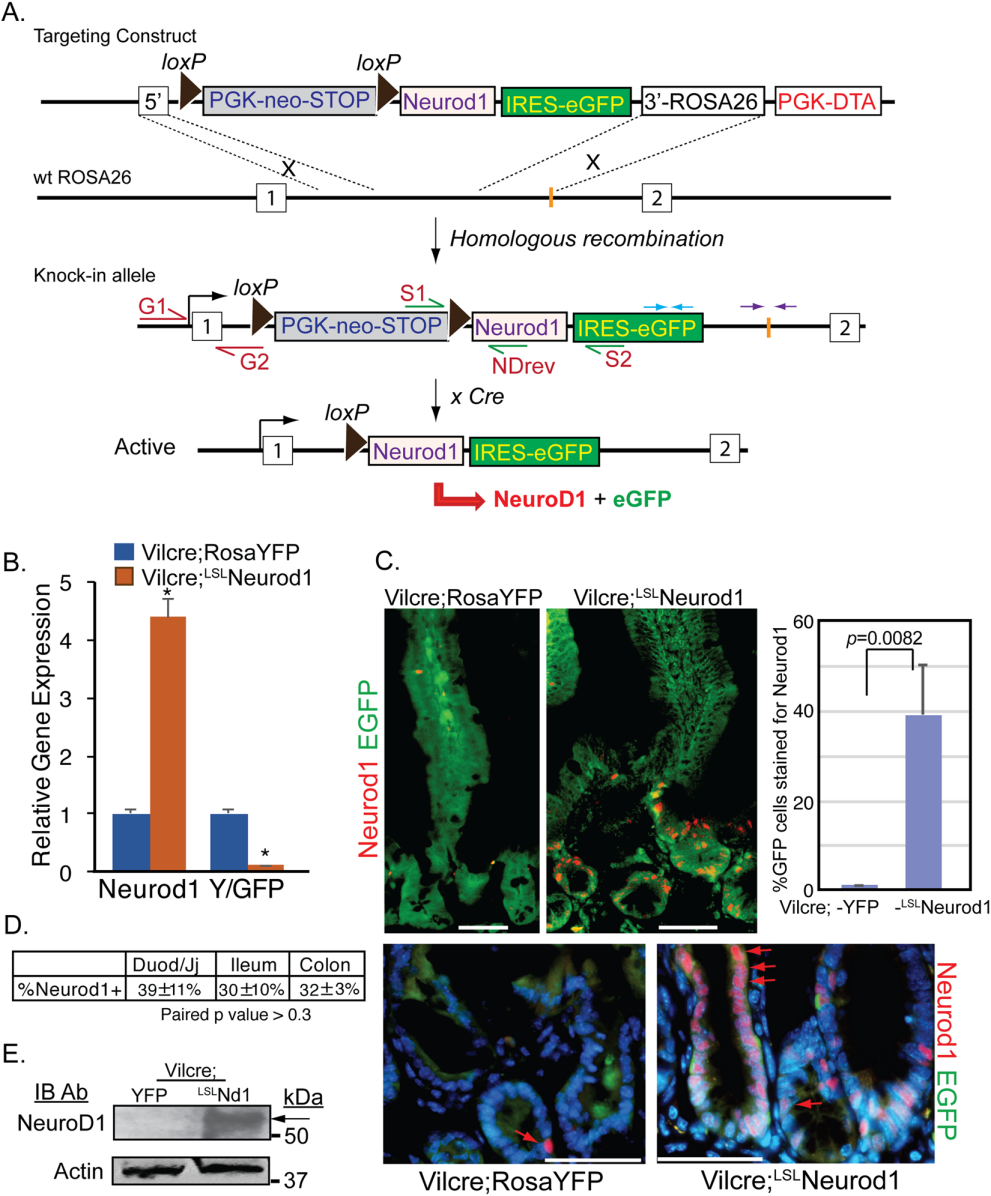
Generation of a conditional Neurod1 mouse line at the ROSA26 locus. (**A**) In ROSA26 loci, the insertion of a loxP-PGK-neo-3xpA (STOP)-loxP sequence upstream of a knock-in Neurod1-IRES-eGFP gene prevents Neurod1 from being transcribed. Cre-mediated deletion of the STOP sequence upon crossing with a driver-mice (e.g, Villin-Cre mice), allows the ROSA26 promoter to drive expression of the Neurod1-IRES-eGFP bi-cistronic fusion transcript. PCR primers used for verification of the indicated locations of recombined clone are shown by arrows of different colors, e.g. red arrows (G1:G2 primers); green arrows (S1:S2 and S1:NDrev); and blue and purple arrows. (**B**) Expression of Neurod1 message is relatively high in Vil-cre;^LSL^ND mice. Relative mRNA levels of Neurod1, EYFP or EGFP (normalized to β-actin) from indicated cells were shown. Values are mean ± SD (n = 3); *P ≤ 0.0001. (**C**) Representative double immunostaining for EYFP/EGFP (green) and Neurod1 (red) of duodenum tissues from Vil-cre; ROSA-YFP and Vil-cre;^LSL^Neurod1 mice. Bar graph shown summarized the %GFP+ cells stained for Neurod1 from indicated mouse intestine. *p* = 0.0082 as indicated. Scale bars = 50 µm. Red arrows pointed to nuclear Neurod1 staining. (**D**) Table summarized the % Neurod1+ cells along intestinal tract. ±represent standard deviation from at least three experimental replicates. The p values between each pair are: 0.45 (Duod to Colon), 0.72 (Ileum to Colon) and 0.33 (Duod to Ileum). These p values are >0.05, indicating no significant difference among the three testing groups. (**E**) Expression of Neurod1 protein in Vil-cre;^LSL^Nd1 mice. Equal amounts of total protein from cell lysates of the duodenum of Vil-cre;YFP and Vil-cre;^LSL^Neurod1 mice (top panel: 60 ug protein/lane; bottom panel: 20 ug protein/lane) were separated on SDS-PAGE followed by immune-blotting with Neurod1 antibody (top panel, black arrow) or Actin antibody (bottom panel).

To test if Neurod1 can switch cell fate and promote EEC differentiation in the developing intestinal epithelium, we generated a conditional gain-of-function Rosa26-^loxp-Stop-loxp(LSL)^Neurod1-IRES-eGFP mice line. A 1,280 bp full-length mouse Neurod1 cDNA was cloned into the pEntry vector (Gateway^**®**^, Life Science). The conditional Rosa26^LSL^Neurod1 targeting vector was created through recombination of the pROSA26-DV1 destination vector with pENTR-mNeurod1 and targeted to the *Rosa26* locus by electroporation into JM8F6 (C57BL/6) mouse embryonic stem (ES) cells^48^. We identified 7 out of 36 correctly targeted ES clones by PCR analysis using primers that cover both *Rosa26* genomic sequence and the inserted transgenes (Fig. 1A). Clone A1 was chosen for C57BL/6 blastocyst injection to generate chimeras for germline transmission. ROSA-^Loxp-STOP-loxp^Neurod1 (^LSL^Neurod1) conditional mice were born viable and fertile.

To determine whether ^LSL^Neurod1 mice can be used to induce Neurod1 expression by Cre-mediated recombination in the small intestine, we crossed ^LSL^Neurod1 mice with Villin-cre driver. Villin is expressed in most intestinal epithelial cells beginning at E13.5^49,50^. Villin-cre-mediated excision of the Loxp-STOP-Loxp sequence allowed transcriptional read-through between the two LoxP sites in the Neurod1-IRES-eGFP bi-cistronic transgene, resulting in Neurod1 and EGFP expression. The expression of Neurod1 and EGFP transgenes were determined by qPCR for transcripts, and immunofluorescent microscopy and immunoblotting for Neurod1 protein. As shown in Fig. 1B, compared with control Vil-cre;^ROSA^EYFP (^LSL^EYFP) mice, Neurod1 transcript in intestinal epithelial cells increased ~4.4-fold in Vil-cre;^LSL^Neurod1 mice whereas EGFP expression level is much lower than that of Vil-cre;^LSL^EYFP mice (11%). This is consistent with earlier findings that genes downstream of IRES are expressed at lower levels compared with the genes upstream of the IRES^51,52^.

The Neurod1 and EG/YFP proteins in the intestine were detected by co-immunostaining with Neurod1 antibody and EGFP antibody which recognized both EGFP and EYFP proteins on duodenal tissues from Vil-cre;^LSL^EYFP and Vil-cre;^LSL^Neurod1 mice (Fig. 1C). As expected, we observed the green EYFP staining throughout crypt-villus structure of the intestine and sporadic Neurod1 nuclei staining (red arrow) in control Vil-cre;^LSL^EYFP mice (Fig. 1C, top left panel). In Vil-cre;^LSL^Neurod1 mice, we observed a significant increase in nuclear Neurod1-positive cells along the intestinal epithelium, mostly in the crypts from the duodenum (39% ± 11%) to the colon (32% ± 3%) (Fig. 1D). Approximately 10% of the Neurod1-positive cells appeared as epithelial ribbons in the crypt (Fig. 1C, bottom right panel). EGFP expression can be detected in both crypt and villi of those cells co-stained with Neurod1. We also analyzed proteins in whole cell lysates from Vilcre;^LSL^Neurod1 mouse intestine that are recognized by the Neurod1 antibody in a Western blotting analysis (Fig. 1E). The antibody detected a single protein band (molecular weight of 50 kDa, the size of Neurod1 protein) with little or no background, further consolidating the above Neurod1 immunostaining results (Supplemental Figure).

### Neurod1 expression in Villin+ cells profoundly increases EE cell numbers

**Figure 2:**
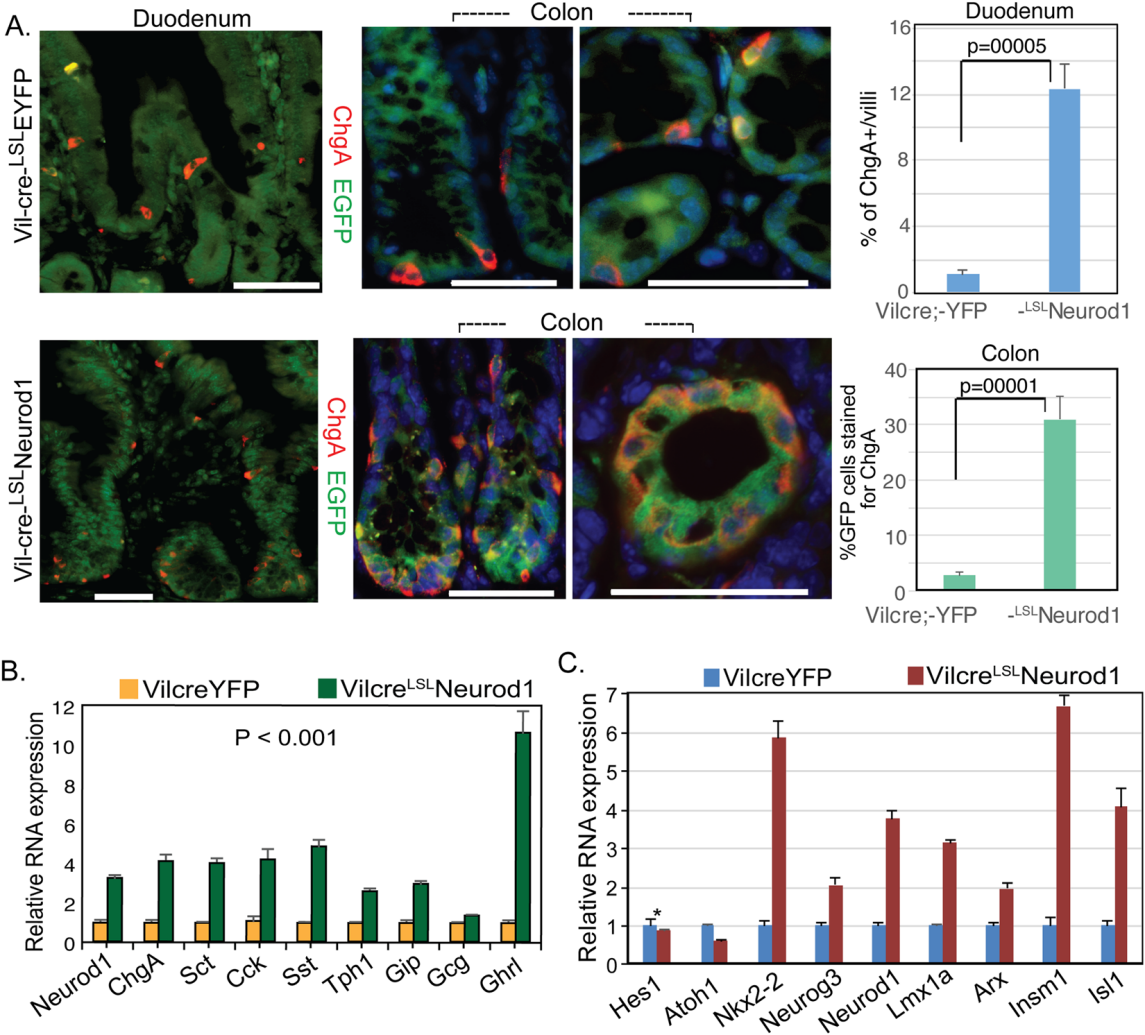
Expansion of EECs with conditional Neurod1 expression in Villin^+^ cells. (**A**) Representative double immunostaining for EYFP/EGFP (green) and ChgA (red) of Crypt and Villus compartments from duodenum and colon samples of Vil-cre;^LSL^EYFP and Vil-cre;^LSL^Neurod1 mice. Bar graph on the right summarized the % of ChgA+ cells in the intestines between two mouse strains as indicated. The p values shown in the graph were: 0.0005 for Duodenum and 0.0001 for Colon. Nuclei were stained with Dapi. Scale bars = 50 µM. (**B**,**C**) Neurod1 in Villin+ cells increased the expression of gastrointestinal hormones (**B**) and EEC-specific transcription factors (**C**). Bar graph represents normalized (normalized to β-actin) mRNA levels of the indicated genes. Values are mean ± SD (n = 3). In B, *P* value of all hormone pairs ≤0.001; in C, *P = 0.21, the rest *P* ≤ 0.001.

To determine if the conditional Neurod1-expressing cells in the intestine of Vil-cre;^LSL^Neurod1 mice were endocrine cells, we stained intestinal sections with a pan-endocrine marker Chromogranin A (ChgA)^53^. A substantial increase in the number of ChgA+ cells in both the duodenal (11.1×) and colonic (11×) epithelium were observed, compared to Vil-cre;^LSL^EYFP control mice (Fig. 2A). In some crypt-villus structures, the increased ChgA+ EGFP + cell number was more pronounced, with nearly all cells in the epithelium expressing ChgA. The increased EE cell population in Vil-cre;^LSL^Neurod1 mice suggested that Neurod1 is able to direct EEC differentiation when ectopically expressed. Contrary to sporadic appearance of EECs in the normal intestine, we observed ChgA+ EECs resided adjacent to each other (Fig. 2A,bottom right) in the intestines of Vil-cre;^LSL^Neurod1 mice, suggesting the loss of lateral inhibition function of Notch signaling by Neurod1, possibly through inhibiting Hes1 function^54^.

We next determined if Neurod1 increased the expression of genes associated with enteroendocrine function and differentiation by qPCR analysis. As shown in Fig. 2B, Neurod1 substantially increased the expression of many EEC hormones or associated genes including secretin (*Sct*), cholecystokinin (*Cck*), somatostatin (*Sst*), tryptophan hydroxylase 1 (*Tph1*), gastric inhibitory polypeptide (*Gip*), and ghrelin; whereas glucagon transcripts did not show much change. The expression of a number of transcription factors associated with endocrine differentiation in the GI tract, including *Nkx2-2, Neurog3, Lmx1a, Insm1, Isl1* and *Arx* were found considerably increased along with Neurod1 (Fig. 2C), consistent with the increase of EEC numbers.

**Figure 3:**
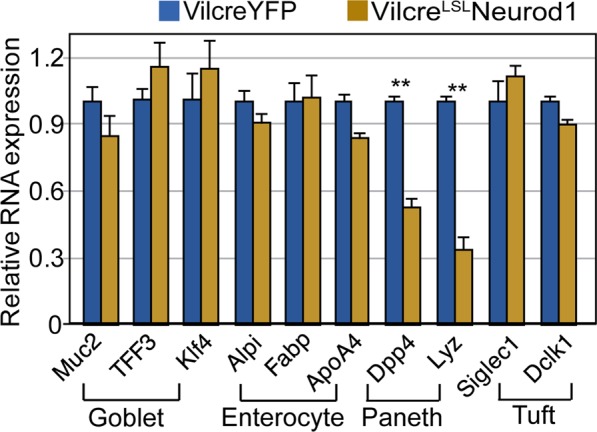
The effects of Neurod1 expression in Villin+ cells on the differentiation of non-EEC cell lineages. Neurod1 in Villin+ cells did not affect marker gene expression for goblet cells and enterocytes. A reduction of Paneth cell marker expression was observed. Bar graph represents normalized (normalized to β-actin) mRNA levels of the indicated genes. Values are mean ± SD (n = 3), **P ≤ 0.0001; No substantial differences were seen of exogenous Neurod1 expression on other lineage marker expression including enterocyte, goblet and tuft cells (*P* ≥ 0.05).

We examined the effects of Neurod1 on the expression of intestinal lineage specific genes including goblet cell (*Muc2, Tff3, Klf4*), enterocytes (*ApoA4, Alpi, Fabp*), Paneth cell (*Lyz1, Dpp4*) and Tuft cell (*Dclk1, Siglec1*) genes. qPCR assay shown in Fig. 3 revealed that the expression of Neurod1 in Villin+ cells had minimal effect on expressions of genes specific for enterocytes, goblet and Tuft cells. However, expression of the Paneth cell specific maker genes *Lyz1* and *Dpp4* were reduced by approximately 60% and 50% respectively. These results suggested that upon Neurod1 expression in Paneth cell progenitors, their maturation is either blocked or shunt towards EEC lineage, possibly via a well-known negative feedback loop of Neurod1 on controlling Atoh1 expression level in developing system^55,56^.

**Figure 4:**
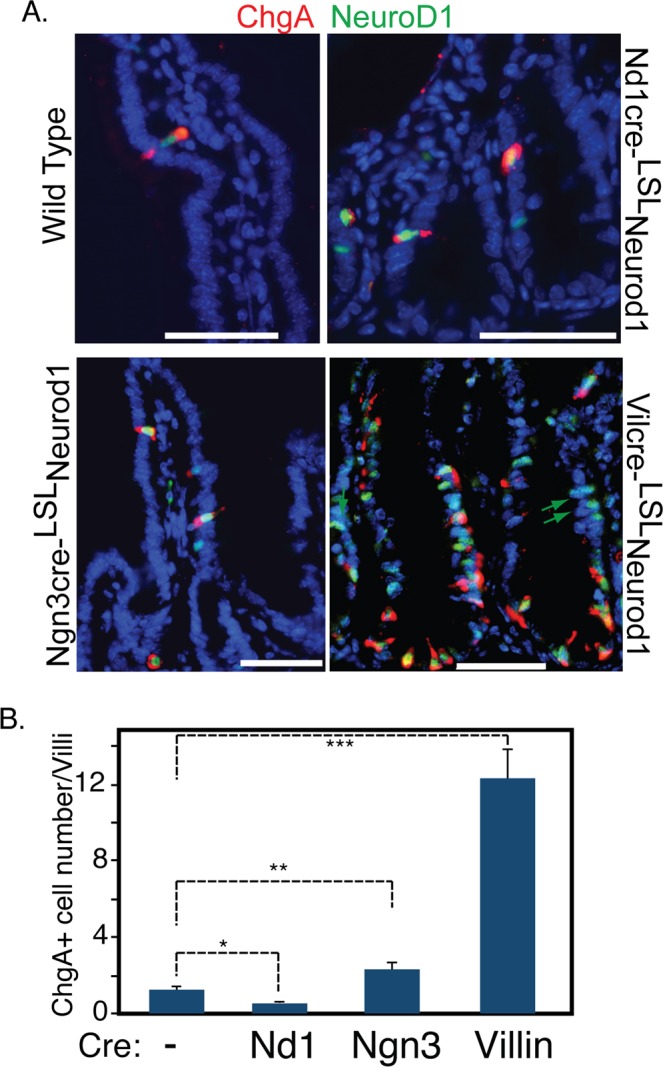
Conditional Neurod1 expression in different EEC differentiation stages. Targeted Neurod1 expression in Neurod1+, Neurog3+ and Villin+ cells were driven by Neurod1-cre, Neurog3-cre and Vil-cre mice respectively. (**A**) Duodenum sections from WT, Neurod1-cre^LSL^Neurod1, Neurog3-cre^LSL^Neurod1 and Vil-cre^LSL^Neurod1 were stained with antibodies against ChgA (red) and Neurod1(green). DAPI (blue) stains for nuclei. Scale bar = 50 µM. (**B**) The number of ChgA^+^ cells in the intestines of indicated Cre-^LSL^Neurod1 mice were quantified as shown in the bar graph. Error bars represent standard deviation from at least three experimental replicates. *P = 0.007; **P = 0.004, ***P ≤ 0.0001.

However, not all Neurod1 expressing cells were ChgA+ (Fig. 4A, green arrow). We observed a 54.4% ± 8% of cells stained for Neurod1 that co-stained with ChgA in the intestines of ROSA26-^LStopL^Neurod mice strain.

### Neurod1 induction of the endocrine lineage depends on the differentiation stage

All five cell types in adult intestinal epithelium are constantly regenerated from multipotential ISCs. The expression of Neurog3 represents one of the earliest stages in the initiation of EEC specification whereas the subsequent expression of its target, Neurod1, occurs at a later stage of EEC differentiation when cells are restricted to an EEC cell fate^7^. To determine the effect of Neurod1 on cell fates in Neurog3+ early EEC progenitors or Neurod1+ late EEC precursors, we crossed ROSA-^LSL^Neurod1 mice with Neurog3-cre or Neurod1-cre mice respectively. As shown in Fig. 4, when Neurod1 expression was activated in Neurog3+ progenitor cells by the Neurog3-cre driver, we observed a small increase (~2 fold) in ChgA+ cell numbers. However, ectopic Neurod1 expression in Neurod1+ cells resulted in a 58% reduction of ChgA+ EEC numbers (Fig. 4B). This suggested a potential function of Neurod1 in promoting EEC differentiation and that may depend on the competency of the target progenitor cells.

### Neurod1 expression in Neurog3 cells blocks their fate towards paneth cells

**Figure 5:**
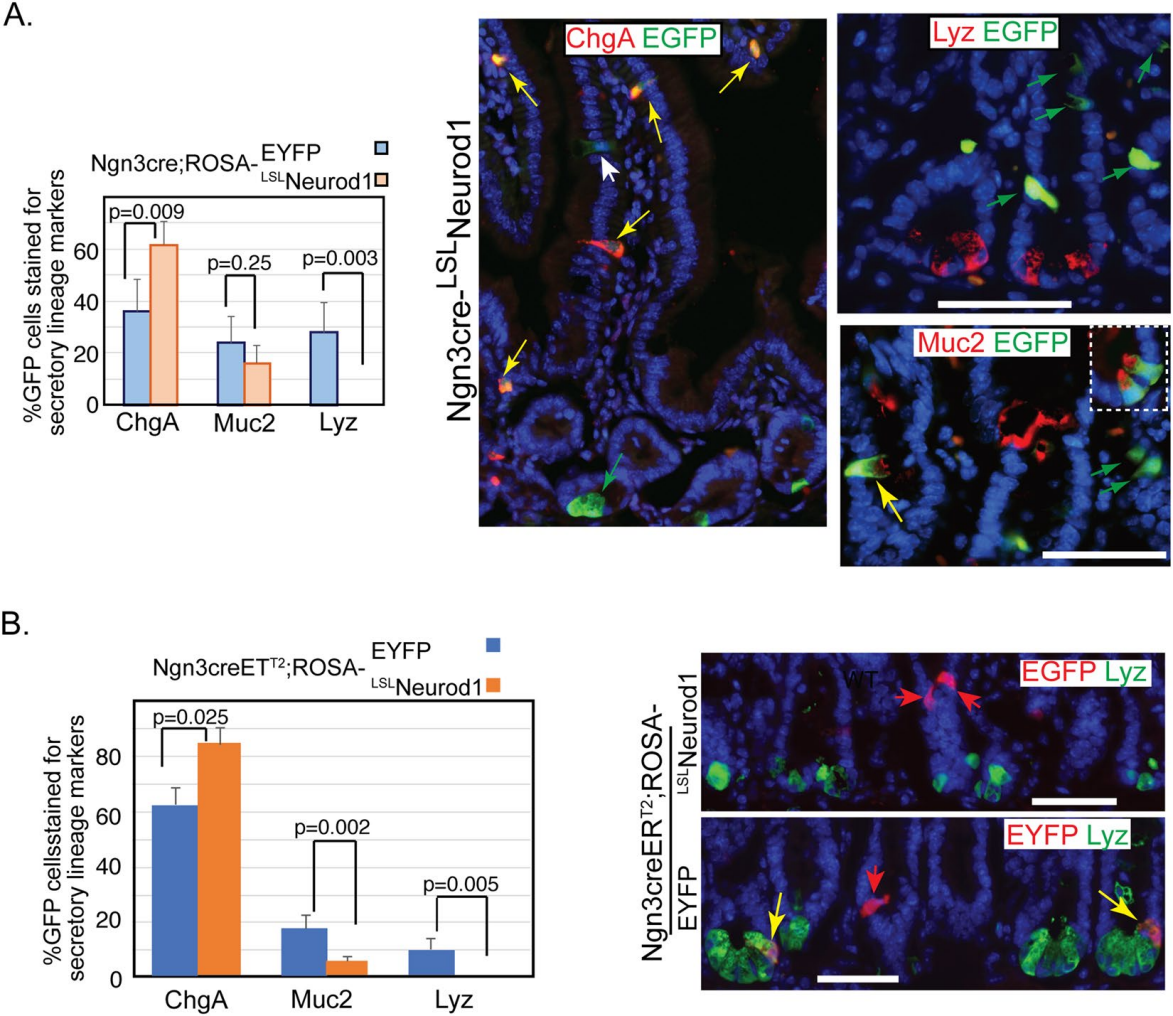
Ectopic expression of Neurod1 in Neurog3+ cells in the intestine prevents Neurog3+ progenitor cells from becoming Paneth cells. Co-Immunofluorescent staining detected EGFP and the Paneth cell marker, lysozyme (Lyz), or goblet cell marker (Muc2) in the duodenum tissues of either Neurog3-cre-^LSL^Neurod1 (**A**) or Tamoxifen-inducible Neurog3-creER^T2^;^LSL^Neurod1. (**B**) Scale bars represent 50 µm. Bar graphs on the left summarized the % GFP+ intestinal epithelial cells stained for EEC (ChgA), Goblet (Muc2) and Paneth (Lyz) cells. Error bars represent standard deviation from at least three experimental replicates. P values were indicated in the graph.

We further analyzed the cell fates of Neurod1+ expressing cells in the intestine of Neurog3-cre;^LSL^Neurod1 mice. Unlike Neurog3+ cells in Neurog3cre;ROSA-EYFP mice that give rise to all three types of secretory cells, including Paneth cells (~30%), goblet cells (~25%), and EEC cells (~40%)^57^, the Neurod1 expressing Neurog3^+^ cells in Neurog3-cre-^LSL^Neurod1 mice gave rise only to EEC and goblet cells (Fig. 5A, yellow arrow). Tuft cells, another secretory cell type present in the small intestine, differentiate from an Atoh1-independent origin^8^. We observed an increase of ChgA+ EEC cell numbers (77% for Neurog3cre;^LSL^Neurod1 and 36% for Neurog3cre^ERT2^; ^LSL^Neurod1 respectively). The Muc2 + EGFP+ goblet cell numbers did not show significant difference upon conditional Neurod1 expression in Neurog3 progenitor cells (p = 0.25, Fig. 5A). We also crossed ROSA-^LSL^Neurod1 mice with the Neurog3-cre-ER^T2^ inducible cre driver line to temporally induce Neurod1 expression in Neurog3+ cells in adult mice. We observed a 35.5% increase in ChgA+ cells (p = 0.025), whereas a significant reduction of goblet cell number was seen (p = 0.002, Fig. 5B). The discrepancy of Neurod1 effect on goblet cell differentiation may depend on the timing of Neurod1 expression.

However, we did not observe EGFP-expression in Lyz^+^ Paneth cells (Fig. 5A). Consistent with what we observed in Neurog3-cre; ^LSL^Neurod1 mice, we did not detect the presence of EGFP + Lyz + cells in the induced adult intestines (Fig. 5B). These data suggested that conditional Neurod1 expression in Neurog3 + secretory progenitors blocked their differentiation to Paneth cells in both developing and adult mice.

## Discussion

Here we generated a new Neurod1 gain-of-function mouse line that enables conditional expression of Neurod1 in Cre expressing cells at different stages of EEC maturation. Whether the intestinal cells would respond to ectopic Neurod1 expression has not been examined. We reported here a substantial increase in the EEC numbers when Neurod1 was conditionally expressed in Villin-Cre+ intestinal cells. Expression of Neurod1 in Neurog3-Cre+ cells only led to a modest increase in EEC numbers whereas reduction in EEC numbers was seen in Neurod1-Cre+ cells. As with the recent findings of Neurod1 as a pioneer factor in the nervous system, our data suggest Neurod1 may function similarly in EEC differentiation and the effects are dependent on differentiation stage.

Sequential expression of bHLH transcription factor Neurogenin 3 and its target Neurod1 is a hallmark of enteroendocrine (EE) lineage specification and maturation. We first considered Neurog3+ EEC progenitors since Neurog3+ cells are multipotent early EEC progenitor cells^46,57^ that give rise to EEC, Goblet and Paneth cells, although majority of Goblet and Paneth cells arise from Gfi+ secretory progenitors. We observed that the expression of Neurod1 resulted in only ~2-fold increase of EEC numbers compared to WT, with no Neurod1 or ChgA epithelial ribbon staining. The increased EEC numbers may be from those Neurog3+ cells that fate to Paneth cells. Our data also suggested that EEC and Paneth cells arise at least in part from common progenitors, in line with recent lineage tracing experiments^46^.

Early lineage tracing in Villin-cre/ROSA26EGFP mice detected wide expression of EGFP throughout the whole intestinal epithelium, including all epithelial cells from intestinal stem cells at the crypt base and all types of matured epithelial cells. However, in the intestine of Villin-cre/^LSL^Neurod1 mice, approximately 30% to 39% of epithelial cells stained for Neurod1 (Fig. 1D), mostly detected in cells located in the crypt and lower villus (Fig. 1C). This may be due to the shorter half-life of Neurod1 leading to decreased Neurod1 along crypt-villus structure. The sensitivity of Neurod1 antibody used in Immunostaining may not be able to detect low levels of Neurod1. Neurod1+ ribbon structure can only be seen in ~10% of the whole intestine. A number of factors may contribute to such a discrepancy on the expression of transgenes between Vil-cre/^L-Stop-L^Neurod1 and Vil-cre/ROSA26R. Recent studies have shown that floxed alleles have different sensitivities to Cre-mediated recombination^58^. The recombination efficiency between different reporter alleles varies greatly on the nucleotide sequences flanking the LoxP sites, distance between loxP sites, the chromosomal location of floxed alleles and the levels of Cre activities.

Unlike normal EECs present as individual mucosal endocrine cells, the ChgA + epithelial ribbon structure in the intestines of Vil-cre+; ^LSL^Neurod1 mice suggested certain Villin + Neurod1+ cells are stem cells. The Intestinal cell types including EECs arise from intestinal stem cells (ISCs) located at the crypt base. The existence of two stem cell pools in the intestinal crypt is well recognized: actively Lgr5+ cycling columnar basal cells (CBCs) and quiescent Bmi1+ +4 position slow cycling stem cells^59,60^. Bmi1+ cells have been suggested as pre-terminal enteroendocrine cells^61^. Intestinal stem cells in each pool are highly heterogeneous. In addition, other slow cycling stem cells have been identified to express other ISC markers such as *Lrig1*^62^, *Hopx*^63^, *mTert*^64^. The identities and relationship of each type of stem cells with distinct ISC markers are not completely clear even after single-cell RNA sequencing of some progenitor pools that has defined hierarchical classification of some subpopulations^46^. Further studies to express Neurod1 in mouse intestine using different progenitor and stem cell marker specific Cre drivers such as *Atoh1*^65^, *Lgr5*^66^, *Bmi1*^60^ and *Lrig1*^62^ may shed more light on the identities of those Neurod1 susceptible early progenitor cells.

Similar to myogenic determining factor *MyoD* that trans-differentiate fibroblasts into functional skeletal muscle cells^67–69^, Neurod1 is able to direct neuron conversion from human cultured fibroblasts^23^ or *in vivo* endogenous mouse astrocytes in the brain and spinal cord^70,71^ when exogenously expressed. The interaction of Neurod1 with Atoh1 plays a role in neurosensory cell differentiation during inner ear development and regeneration^37,72,73^. The potential of Neurod1 to induce differentiation - including fate switching - in immature cells, suggests that it could play a major role in direct differentiation of cells, short-circuiting the current attempts to generate differentiated cells out of induced pluripotent stem cells. Consistent with this idea, recent studies showed that Neurod1 can function as a pioneer factor and its expression in microglia can directly convert microglia to functional neurons in the adult striatum^47^. Microglial cells are extremely plastic; it can undergo a variety of structure changes upon different stimuli. Like other pioneer factors, Neurod1 induces global transcriptional changes when exogenously expressed in microglia by occupying chromatin loci to modulate chromatin structure. Our preliminary results using a ChIP-seq assay identified over 7,500 substantial Neurod1 occupied loci in STC neuroendocrine cells (https://doi.org/10.1016/S0016-5085(17)30723-0), consistent with recent publication showing that nearly 3000 loci were regulated by Neurod1^74^. Taken together, our data suggest a broader role of Neurod1 as a possible pioneer factor in EEC differentiation.

We previously showed by lineage tracing using Neurog3-cre;ROSA-EYFP mice that Neurog3+ cells gave rise to not only EEC, but also Goblet and Paneth cells^57^. Here we found that the gain-of-function of Neurod1 expression in Neurog3+ cells blocked Neurog3+ cells from differentiating into Paneth cells (Fig. 5). Similarly, when Neurod1 was expressed in Villin+ cells in Vilcre;^LSL^Neurod1 mice, we observed a considerable decrease in Paneth cell marker expression and no effect to Goblet and Tuft cell markers (Fig. 3). These results suggest that Neurod1 expression restricts intestinal epithelial cells towards EECs inhibiting Paneth cell lineage.

The canonical Wnt signaling pathway plays an important role not only in stem cell maintenance but also in Paneth cell differentiation and maturation processes^75^. Conditional expression of non-degradable mutant beta-catenin (exon3 deletion) in Neurod1+ EECs failed to activate wnt pathway, while the wnt activation occurred when the same mutant beta-catenin was expressed in Neurog3+ cells^76^. The onset of Neurod1 expression resulted in the failure of Wnt activation and inhibition of its target gene c-Myc expression^76^. In fact, a number of tissue specific transcription factors have been described to interact with β-catenin and inhibit Wnt activation in a number of developmental systems including chondrogenesis and hematopoiesis^77–83^. Those interactions lead to blocking of differentiation through disrupting β-catenin/TCF4 complex (*Icat, Hif1a, Cdx2*) or its binding to DNA (*Runx3, Pdx1, Hbp1*) or preventing coactivators recruitment (*Klf4*). Neurod1 might adopt similar mechanism in inhibiting Wnt activation. This may represent a novel yet unappreciated role of Neurod1 in mediating transcriptional repression of target gene expression.

## Methods

### Mice

The Institutional Animal Care and Use Committee (IACUC) at the University of Massachusetts Medical School approved all experimental protocols of our vertebrate animal studies in accordance with National Institute of Health (NIH) guidelines. All mice were housed in AALACC certified facilities, under constant temperature, air pressure and specific pathogen-free conditions and a 12-hour light cycle.

For lineage tracing experiments, we used previously described Cre-driver mouse lines including Vil-cre^49^, Neurog3-cre^57^, Neurog3-cre^ERT2^ and Neurod1-cre^84^. To induce Cre activity in Neurog3cre^ERT2^ compounded mice, 4-week old mice were treated with 2 mg tamoxifen (Sigma, T5648) dissolved in corn oil with one intraperitoneal injection daily for 5 consecutive days. Tissues were harvested and analyzed 7 days later.

### Targeting loxp-neurod1-loxp ES cells

A pEntry-mNeurod1 plasmid was generated by cloning full-length mouse Neurod1 cDNA into the pEntry vector (Gateway). A Gateway-compatible pROSA26 Destination Vector (pROSA26-DV1 LMBP 6350)^48^ was used to generate a Rosa26 locus targeting vector for conditional overexpression of Neurod1 with an ires-EGFP reporter. Clones were verified by both enzymatic digestion and sequencing. A linearized ROSA26-DV1-mNeurod1 plasmid was electroporated into C57BL/6 derived JM8.F6 ES cells^85^. G418-resistant ES-cell clones were screened by PCR using primers set at both 5′ ROSA26 and 3′ covering loxP site: G1: 5′-TAGGTAGGGGATCGGGACTCT-3′; and G2: 5′-GCGAAGAGTTTGTCCTCAACC-3′ to generate a 1.3 kb fragment. PCR-positive clones were further verified with additional PCR reactions using both Neurod1 internal and external ROSA-3′UTR primers and EGFP primers as indicated in Fig. 1: S1: ATCATGTCTGGATCCCCATC; S2: GGGGCGGAATTCGATATCAAG. mND552rev: TGGTAGTGGGCTGGGACAAACCTTT. Rosa3′UTR-DV1-for: AACAGAGGCTGTTGGTACTAGTGGC; Rosa3′UTRrev: AGCACCAAATGTGGTGCAGT. EGFP-for: AAG TTC ATC TGC ACC ACC G; EGFPrev: TCC TTG AAG AAG ATG GTG CG.

### Generation of chimeras

The chimeras were generated by our Transgenic Core facility (https://www.umassmed.edu/tkomouse/). In brief, obtained positive JM8.F6 ES cell clones were injected into albino C57BL/6J_tyrc-Brd_ blastocysts. The resulting chimeras were bred with Albino C57BL/6J_tyrc-Brd_ mice. Germline transmitted transgenic mice that carried targeted allele were selected by fur color and confirmed by genotyping using PCR analysis of DNA isolated from mice ear snips. Primer pairs S1:mND552rev shown in Fig. 1 were used in the PCR reaction.

### Intestinal epithelial cell isolation

Mouse intestinal crypt and villus isolation were performed according to a published protocol with modifications^86^. In brief, mice were euthanized by CO_2_ and cervical dislocation. The small intestines were flushed with ice-cold 1xPBS and everted on a 4 mm rod. Intestinal tissues were cut into 2 cm pieces and incubated in chelating buffer [pH = 7.3: 1xD-PBS (1 mM CaC_l2_, 0.5 mM MgC_l2_, 8 mM Na_2_HPO_4_, 137 mM-NaCl, 1.5 mM-KH_2_2PO_4_, 2.7 mM-KCl), 1 mM-DTT, 1 mM EDTA] at 4 °C with constant shaking for 30 min. The cells were released as large sheets of villus cells. The tissues were transferred to fresh chelating buffer and were vigorously shaken to release crypts into the media. The collected villus and crypt portions were filtered through a 70 µm Cell Strainer and pelleted at 200 rpm for 5 minutes with low brake speed. The villus and crypt pellets were resuspended in Trizol (Invitrogen, Inc.) for RNA extraction.

### RNA isolation and qPCR analysis

RNAs were prepared with RNeasy Mini kit (Qiagen). In-column DNA digestion was performed to remove possible DNA contamination using RNase-free DNase (Qiagen). cDNAs were prepared using the Maxima First-Strand cDNA synthesis kit for RT-qPCR (Thermo Scientific). qPCRs were performed according to standard protocol using the Light Cycler (BioRad). Results are given as relative expression normalized to housekeeping gene beta-actin. Primer sequences are available upon request.

### Immunofluorescence staining and western blotting

Intestinal tissues were fixed in 4% formaldehyde and embedded in OCT for frozen sections. For immunofluorescence analysis, the following primary antibodies were used: goat anti-EGFP (1:400, Novus Biologicals: NB100-1770), rabbit anti-Chromogranin A (1:1000, Immunostar: #20085), rabbit anti-Muc2 (1:500, Santa Cruz: H-300), and rabbit anti-Lyz (1:500, Zymed Laboratories: 18-0039), rabbit anti-Neurod1 (1:250, Abcam: Ab109224). Alexa Fluor 488 or 594 conjugated secondary antibodies were used at a dilution of 1:800. Intestinal Alkaline Phosphatase activities were detected according to the manufacturer’s protocol (Vector Red AP Substrate Kit, Cat. #: SK-5100). Images were aquired using a Nikon Eclipse E600 microscope with imaging software of NIS-Elements AR 4.30.02.

Western blotting was performed as previous describe^22^. In brief, equal amount of proteins was separated by SDS-PAGE and eleterotransferred onto a nitrocellulose membrane. Nonspecific binding to the membrane was blocked with a 3% milk solution in phosphate-buffered saline (PBS) containing 0.1% NP-40 prior to incubation with an anti-Neurod1 antibody (1:1,000; Abcam clone #Ab109224) or anti-actin antibody (1:2,000, Santa Cruz: sc-1616 HRP). An enhanced chemiluminescence (ECL) kit (Amersham) was used for detection of protein.

### Statistical analysis

Pair-wise comparisons were analyzed by the Student’s *t* test. The statistical significance of pairwise comparisons shown on the bar graph were indicated with *P* ≤ 0.05 as significant and P > 0.05 as non-significant.

## Supplementary information

Supplemental Figure

## Data Availability

No datasets were generated or analyzed during the current study.
