# Supplementary material for: Intestinal Neurod1 expression impairs paneth cell differentiation and promotes enteroendocrine lineage specification
Source: Sci Rep. 2019 Dec 20;9:19489. doi: 10.1038/s41598-019-55292-7 (PMC6925293; doi:10.1038/s41598-019-55292-7)
Supplement: Supplementary file 1 — Supplemental Figure [file 41598_2019_55292_MOESM1_ESM.pdf]

Intestinal Neurod1 expression impairs Paneth cell differentiation and promotes enteroendocrine lineage specification

Hui Joyce Li, Subir K. Ray, Ning Pan, Jody Haigh, Bernd Fritzsch, and Andrew Leiter*

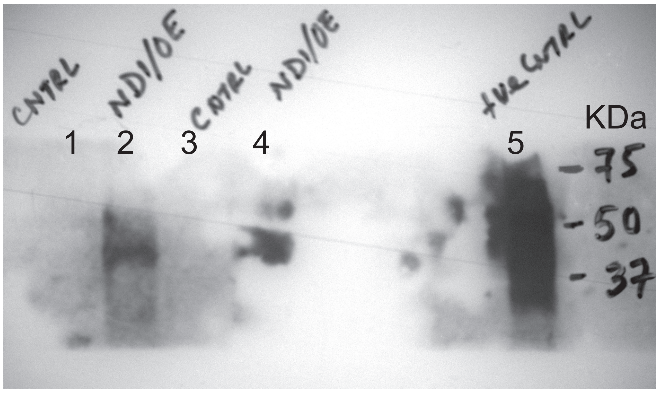

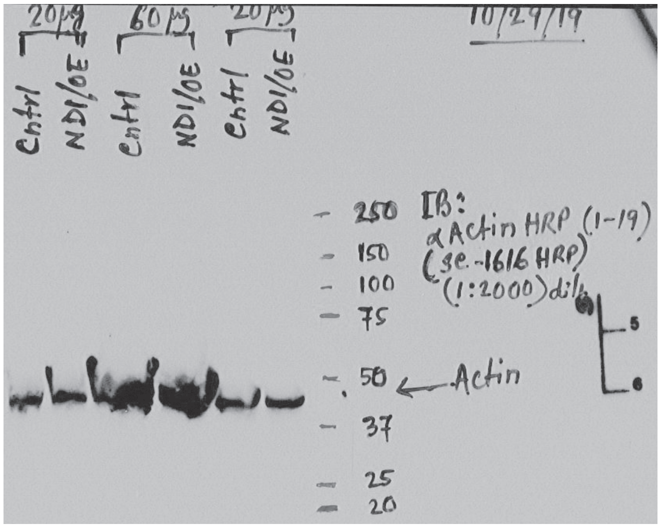

Shorter exposure

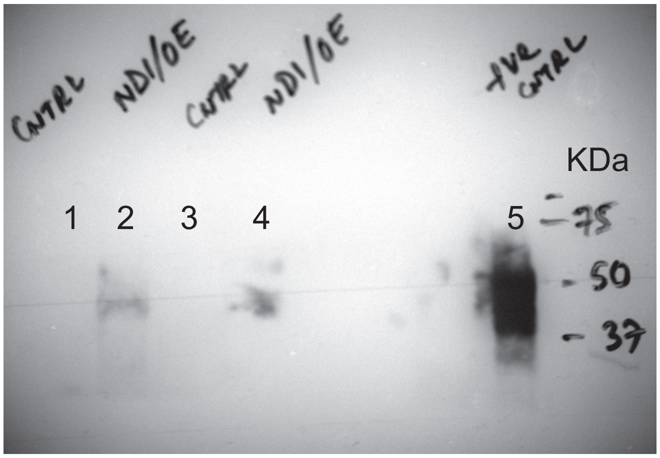

CNTRL = control;
ND1/OE = Neurod1 overexpression;
+ve CNTRL = positive control

Supplementary Figure. Whole film for western blot in Figure 1E. Whole cell protein extracts from duodenum of control (CNTRL, lane 1 and 3) and Vilcre; ^{LSL}Neurod1 (ND1/OE, lane 2 and 4) mice (60 ug protein for lane 1 and 2; 20 ug for lane 3 and 4) were loaded onto SDS-PAGE and electro-transferred proteins onto the blot. Gel on the left, Lane 5 is 2 ul lysate from in vitro translated Neurod1 protein in rabbit reticulocyte lysates as positive control. We probed a larger portion of the blot, which included proteins ranging from 37 kDa to 75 kDa covering NeuroD1 (47 kDa) with a commercially available recombinant rabbit monoclonal antibody to NeuroD1 (Abcam, clone #ab109224). We used a portion of the blot to conserve antibody for future experiments. This antibody has already been shown to work well for Neurod1 in western blot without background signals [1,2]. Both shorter (bottom panel) and longer exposure (top panel) of the same blot were shown. Gel on the right, the blot was probed with antibody agaist actin (Santa Cruz, sc-1616, 1:2,000).

1 Wolnerhanssen, B. K. et al.. Sci Rep 7, 8174, doi:10.1038/s41598-017-08487-9 (2017).
2 Mollaoglu, G. et al. Cancer Cell 31, 270-285, doi:10.1016/j.ccell.2016.12.005 (2017).
